# Shallow-angle intracranial cannula for repeated infusion and *in vivo* imaging with multiphoton microscopy

**DOI:** 10.1117/1.NPh.12.2.025001

**Published:** 2025-03-25

**Authors:** Steven S. Hou, Joyce Yang, Yeseo Kwon, Qi Pian, Yijing Tang, Christine A. Dauphinais, Maria Calvo-Rodriguez, Mirna El Khatib, Sergei A. Vinogradov, Sava Sakadzic, Brian J. Bacskai

**Affiliations:** a Massachusetts General Hospital, Harvard Medical School, Department of Neurology, Boston, Massachusetts, United States; b Massachusetts General Hospital, Harvard Medical School, Athinoula A. Martinos Center for Biomedical Imaging, Boston, Massachusetts, United States; c University of Pennsylvania, Department of Biochemistry and Biophysics, Philadelphia, Pennsylvania, United States; d University of Pennsylvania, Department of Chemistry, Philadelphia, Pennsylvania, United States

**Keywords:** multiphoton microscopy, cannula, cranial window, brain, *in vivo* imaging, fluorescence, chemical sensors, oxygen, phosphorescence, cell death

## Abstract

**Significance:**

Multiphoton microscopy serves as an essential tool for high-resolution imaging of the living mouse brain. To facilitate optical access to the brain during imaging, cranial window surgery is commonly used. However, this procedure restricts physical access above the imaging area and hinders the direct delivery of imaging agents and chemical compounds to the brain.

**Aim:**

We aim to develop a method that allows the repeated administration of imaging agents and compounds to the mouse brain while performing *in vivo* imaging with multiphoton microscopy.

**Approach:**

We have developed a cannula delivery system that enables the implantation of a low-profile cannula nearly parallel to the brain surface at angles as shallow as 8 deg while maintaining compatibility with multiphoton microscopy.

**Results:**

To validate our shallow-angle cannula approach, we performed direct infusion and imaging of various fluorescent cell markers in the brain. In addition, we successfully demonstrated tracking of degenerating neurons over time in Alzheimer’s disease mice using Fluoro-Jade C. Furthermore, we showed longitudinal imaging of the partial pressure of oxygen in brain tissue using a phosphorescent oxygen sensor.

**Conclusions:**

Our developed technique should enable a wide range of longitudinal imaging studies in the mouse brain.

## 1 Introduction

*In vivo* imaging with multiphoton microscopy has become an indispensable technique for studying cortical function in the intact brain.^1^^–^^3^ Due to the opacity of the mouse skull, a crucial step in preparation for multiphoton imaging is the surgical procedure that allows optical access to the brain. In the thinned skull approach, a small portion of the skull is made transparent by carefully drilling down to ∼20  μm.^4^^,^^5^ Although this approach is minimally invasive, the skull regrowth limits its longitudinal application. In the cranial window approach, a bone flap from the skull is completely removed, and a cover glass is sealed over the exposed brain.^6^^,^^7^ Although the cranial window grants optical access to the brain, physical access to the brain is restricted.

The delivery of chemical compounds, including imaging agents and drugs, into the brain is an essential component of many multiphoton microscopy imaging studies. However, many compounds cannot cross the blood–brain barrier (BBB) and, therefore, cannot be delivered to the brain through systemic administration.^8^ To enter the brain, they need to be directly applied, either topically to the brain surface or by injection with a needle or micropipette.^9^ As the cranial window imposes a physical barrier to the brain, compounds often need to be delivered to the brain at the same time as cranial window surgery and before the cover glass is sealed. As further access to the brain is restricted after the cranial window is in place, the ability to repeatedly deliver to the brain and perform longitudinal imaging is not possible using the standard cranial window approach.

Recently, new techniques have been developed that combine direct delivery to the mouse brain with *in vivo* imaging capability. One class of methods uses soft, transparent materials in place of the cover glass for the cranial window.^10^^,^^11^ The softness of the material permits penetration of needles and micropipettes, whereas its transparency is sufficient for multiphoton microscopy. A related technique incorporates silicone access ports within the glass coverslip, enabling injections through these ports while simultaneously allowing for imaging through the cover glass.^12^ Another approach to provide access to the brain has been the development of removable cranial windows.^13^ Here, the cover glass can be removed to grant temporary access to the brain and then resealed. The main drawback of these techniques is that they require the repeated insertion of the injection needle or micropipette into the brain tissue which may lead to tissue damage, bleeding, and inflammation. This can significantly affect the quality of the imaging and impede longitudinal imaging. Furthermore, the removal of the cranial window can cause additional inflammation from recurring brain exposure.

Techniques based on implantable devices have also been used for delivery into the brain. In one approach, a micro-optical fluidic device is created with an inlet and outlet for infusion and removal of chemical compounds.^14^ Although this approach allows repeated application of compounds at the brain surface, the relative amounts of the compound that reaches different depths of the brain parenchyma cannot be varied and are strongly dependent on its diffusion properties. Also, the need for dura removal during implantation of the device could lead to complications after surgery recovery. In another technique, commercially available cannulas are implanted into the brain for delivery, whereas cranial windows are placed adjacent to the cannula.^15^^,^^16^ However, the larger size of these cannulas imposes constraints on the implantation to steeper angles (previous studies have used 45 deg^15^ and 90 deg^16^). For cannulas targeted toward the superficial cortex, the steeper angles result in the cannula outlet location being close to the edge of the cranial window, where imaging can be challenging and only a small part of the imageable brain area may be exposed to the injected substance.

In this study, we developed a custom cannula delivery system that enables the permanent implantation of a low-profile cannula at angles as shallow as 8 deg into the mouse brain. The shallow angle of the implanted cannula allows its tip to be centered over a 4-mm cranial window while still being located within the superficial layers of the mouse cortex. Importantly, the cannula does not interfere with the cranial window or with imaging using multiphoton microscopy. We validated our method using various fluorescent cell markers to confirm successful delivery and imaging capability. In addition, we demonstrated repeated infusion and longitudinal imaging by tracking neurodegeneration in a mouse model of Alzheimer’s disease using Fluoro-Jade C, a fluorophore traditionally employed for staining *ex vivo* tissue sections.^17^ Furthermore, we demonstrated longitudinal functional imaging of tissue partial pressure of oxygen (${\mathrm{pO}}_{2}$) through the direct infusion of the phosphorescent oxygen sensor, Oxyphor 2P,^18^ and *in vivo* phosphorescence lifetime imaging.^19^^–^^21^

## 2 Methods

### 2.1 Animals

We used C57BL/6J mice (The Jackson Laboratory, Bar Harbor, Maine, United States) for validating our cannula-based delivery system using various fluorescent cell markers and for *in vivo* longitudinal ${\mathrm{pO}}_{2}$ imaging. Mice of both sexes at 4 to 6 months of age were used. For the imaging of neurofibrillary tangles and tracking of neurodegeneration, we used rTg4510 [The Jackson Laboratory, Tg(Camk2a-tTA)1Mmay Fgf14Tg (tetO-MAPT*P301L)4510Kh] mice expressing the tetracycline-controlled transactivator protein (tTA)^22^ along with age-matched non-transgenic littermates. Mice of both sexes at 6 to 8 months of age were used. For imaging of amyloid aggregates, we used APP/PS1 [The Jackson Laboratory, B6.Cg-Tg (APPswe, PSEN1-dE9)85Dbo/Mmjax] double-transgenic mice expressing mutant human APPswe/human PS1-ΔE9.^23^ Mice of both sexes were used at 12 to 13 months of age.

Animals were housed in an AAALAC International-certified facility (Massachusetts General Hospital, D16-00361), and all animal studies were performed under the supervision of the Massachusetts General Hospital Institutional Animal Care and Use Committee in accordance with the approved institutional protocol (2018N000131).

### 2.2 Cannula, Dummy Wire, and Holder Design

The custom cannula was constructed by combining a 6-mm length, 26-G stainless steel tubing (6689, New England Small Tube, Litchfield, New Hampshire, United States) with a 9.6-mm length, 33-G stainless steel tubing (33RW-378-45 deg, Vita Needle, Needham, Massachusetts, United States). The tip of the 33-G tubing was beveled at 45 deg. The 26-G tubing was aligned to the 33-G tubing using a custom cannula assembly part, and the two tubings were attached using epoxy (ClearWeld, J-B Weld, Marietta, Georgia, United States). For cannulas used in this study, the cutting, beveling, and assembly were also performed at Protech International. The cannula holder was machined from high-density polyethylene, and a hole was created using a 0.0189-in. drill bit (McMaster-Carr, Elmhurst, Illinois, United States) for securing the 26-G tubing (MGH Model Shop, Boston, Massachusetts, United States). The dummy wire was created using tungsten wires (0.00355-in. outer diameter, Hamilton, Reno, Nevada, United States) with the length matched to the cannula length. The fluorescent dummy wire was created using smaller diameter tungsten wires (0.002-in. outer diameter, Thermo Scientific Chemicals, Waltham, Massachusetts, United States); 10-mM stock solution of SR101 in dimethyl sulfoxide (DMSO) was mixed with an ultraviolet (UV) curable adhesive (NOA61, Thorlabs, Newton, New Jersey, United States). The mixture was applied to the tip of the dummy wire and then cured with a UV LED (M365L3, Thorlabs). The length of the fluorescent dummy wire was matched to the length of the cannula. The schematic for the cannula and design files for the cannula holder and assembly part are available at https://github.com/shou-lab/ShallowAngleCannula.

### 2.3 Surgery and Cannula Implantation

Mice were anesthetized with isoflurane (5% for induction and 1.5% throughout the surgery) using a low-flow anesthesia system (SomnoSuite, Kent Scientific, Torrington, Connecticut, United States) and placed into a custom head holder (MGH Model Shop). Dexamethasone ($0.2\;\mathrm{mg}\;{\mathrm{kg}}^{-1}$) was administered subcutaneously to reduce brain swelling. A heating pad (HTP-1500, Kent Scientific) was used to maintain the mouse’s body temperature during surgery. The scalp was shaved and sterilized. An incision was made to expose the underlying skull. A 1-mm craniotomy was created centered at 2.5 mm lateral to the bregma. To ensure, the cannula holder would not make contact with the skull during the implantation process, a rectangular region of the skull above the 1-mm craniotomy was progressively thinned down, forming a sloped ramp leading to the 1-mm craniotomy. The mouse’s head position in the holder was adjusted so that the skull at the bregma and at 2 mm below the bregma were at the same height. The cannula was inserted into its holder and rotated so that the bevel faced toward the top of the brain after implantation. The cannula holder was attached to the manipulator arm of the mouse stereotaxic (RWD Life Science, Shenzhen, China). The angle of the manipulator arm was set according to the implantation angle (θ). We calculated θ using the equation tan(θ)=depth/2  mm where depth is the target depth in the brain for the cannula tip. The mouse was elevated by placing the mouse head holder onto a custom platform. The cannula tip was brought down to touch the mouse dura at the center of the 1-mm craniotomy. The distance (dist) that the cannula travels in the brain is calculated using the equation dist=sin(θ)/depth. After the cannula is implanted into the brain, the cannula is secured to the skull using dental acrylic (Jet Denture, Lang Dental Manufacturing, Wheeling, Illinois, United States) and cyanoacrylate glue (Krazy Glue). A cyanoacrylate accelerator (Insta-Set, Bob Smith Industries, Paso Robles, California, United States) is applied to quickly harden the mixture, and the holder is retracted. Next, a craniotomy is performed below the location of the implanted cannula by removal of a ∼4-mm piece of the skull. The dura mater was left intact during the craniotomy procedure. A 4-mm-diameter cover glass was placed over the exposed brain and sealed with a mixture of dental acrylic and cyanoacrylate glue. After the cranial window surgery, 0.5  μL of phosphate-buffered saline (PBS) was infused through the cannula, and the polyethylene tubing was cut and sealed with dental acrylic and cyanoacrylate glue (see Sec 2.4). Mice were given buprenorphine ($0.1\;\mathrm{mg}\;{\mathrm{kg}}^{-1}$) and Tylenol for 3 days after surgery and were allowed to recover for at least 3 weeks before imaging experiments. Prior to imaging, an aluminum headplate with a 5-mm observation hole (CP-1, Narishige Group, Tokyo, Japan) was secured to the cranial window using dental acrylic and cyanoacrylate glue.

### 2.4 Cannula Infusion

Mice were anesthetized during the cannula infusion procedure. To prepare for the infusion, polyethylene tubing (PE20, Becton Dickinson, Franklin Lakes, New Jersey, United States) was connected to a 26-G hypodermic needle (Becton Dickinson), and a 25-μL syringe (1702 TLLX, Hamilton) was attached to the Luer Lock connector of the needle. The syringe, needle, and polyethylene tubing were first filled with PBS, and then the tubing was filled with the compound to be infused. A small air gap was introduced between the PBS and compound as a reference to confirm the amount of the compound that entered the brain. To ensure the cannula was clear, the tungsten dummy wire was inserted into and removed from the cannula several times. Then, the open end of the polyethylene tubing was attached to the cannula. A syringe pump (NE-300, New Era Pump Systems, Farmingdale, New York, United States) was used to push the syringe for the infusion. The infusion rate for imaging experiments was 0.05  μL/min. Following infusion of the compound, 0.5  μL of PBS was infused to clear the cannula of the compound. The polyethylene tubing was then cut, and a small drop of dental acrylic and cyanoacrylate glue was applied to the end of the tube to seal it.

### 2.5 *In Vivo* Multiphoton Microscopy

*In vivo* fluorescence imaging was performed using a commercial multiphoton microscope (FV1000MPE, Olympus, Tokyo, Japan). The microscope was equipped with a Ti:sapphire laser (Mai Tai HP DeepSee, Spectra-Physics, Milpitas, California, United States) with a tuning range of 690 to 1040 nm. A 25× water immersion objective (XLPLN25XWMP2, NA = 1.05, Olympus) was used to acquire images. Fluorescence emission was collected in the blue (420 to 460 nm), green (495 to 540 nm), and red (575 to 630 nm) spectral channels. During imaging, mice were anesthetized, and body temperature was regulated using a heating pad (Kent Scientific). To stabilize the mouse head for imaging, mice were placed into a headplate holder (MAG-1, Narishige Group). The following volumes and concentrations were used for the labeling of various cell types and structures in the brain: 6  μL of 89.0  μM Hoechst 33342 for labeling the cell nuclei, 1  μL of 1:5 dilution of stock NeuroTrace 500/525 for labeling the pericytes, 1  μL of 20  μM SR101 for labeling the astrocytes, 1  μL of 100  μg/mL Isolectin GS-IB4, Alexa Fluor 594 conjugate for labeling the microglia, 1  μL of 100  μM LysoTracker Green DND-26 for labeling the lysosomes, 1  μL of 100  μg/mL fluorescein-labeled *Wisteria floribunda* lectin (Vector Laboratories, Newark, California, United States) for labeling the perineuronal nets (PNNs), 2  μL of 1  mg/mL HiLyte Fluor 488-labeled Aβ42 (Anaspec), 1  μL of 100  μg/mL ThioS (Sigma Aldrich, St. Louis, Missouri, United States) for labeling the amyloid plaques, and 2  μL of 5  mg/mL Thiazine Red (Sigma Aldrich) for labeling of the neurofibrillary tangles (NFTs). For angiograms, 100  μL of either 12.5  mg/mL fluorescein dextran, 70 kDa, or 12.5  mg/mL rhodamine B dextran, 70 kDa was injected intravenously. Fluorescent cell markers were purchased from Thermo Fisher Scientific unless otherwise specified.

### 2.6 *In Vivo* ${\mathrm{pO}}_{2}$ Imaging Using Phosphorescence Lifetime Imaging Microscopy

*In vivo* ${\mathrm{pO}}_{2}$ images were acquired using a custom multiphoton microscope equipped with a tunable pulsed laser (Chameleon Discovery NX, Coherent, Saxonburg, Pennsylvania, United States) (100-fs pulse width and 80-MHz repetition rate). Its output power was modulated by an electro-optic modulator (350-105-02, Conoptics, Danbury, Connecticut, United States) to generate repeated 300-μs laser excitation cycles (10-μs laser on time; 290-μs laser off time) for ${\mathrm{pO}}_{2}$ imaging or constant average power for angiogram imaging. Lateral imaging was achieved by scanning the laser beam using a two-axis galvanometer mirror system (6210H, Cambridge Technology, Bedford, Massachusetts, United States) with the corresponding scan lens (∼51-mm focal length, 2× AC508-100-B, Thorlabs) and tube lens (180-mm focal length, Olympus). Axial imaging was conducted by moving the objective using a piezo objective scanner (PFM450E, Thorlabs). A 25× water immersion objective (XLPLN25XWMP2, NA = 1.05, Olympus) was used to focus the excitation beam inside the sample. A dichroic mirror (FF875-Di01-38.1X51, Semrock, Rochester, New York, United States) was used to separate the laser excitation and the emission signals. The emission signals were further filtered by several filters and dichroic mirrors for ${\mathrm{pO}}_{2}$ imaging (FF01-890/SP-50, FF560-Di01-35X52, and FF01-795/150-25, Semrock) and angiogram imaging (FF01-890/SP-50, FF560-Di01-35X52, and FF03-525/50-25, Semrock) before they were detected by two photomultiplier tubes (PMTs) (${\mathrm{pO}}_{2}$: H10770PA-50, Hamamatsu, Shizuoka, Japan; angiogram imaging: R3896, Hamamatsu, respectively). ${\mathrm{pO}}_{2}$ signals were collected using a photon counting unit (C9744, Hamamatsu) and a digital counter (PXIe6341, National Instruments, Austin, Texas, United States), whereas the acquisition of angiograms was facilitated by a preamplifier (C9999, Hamamatsu) and an analog input channel (PXIe6356, National Instruments). The whole imaging process was completed under the control of a custom MATLAB software (MathWorks, Natick, Massachusetts, United States). The temperature of the water immersion was maintained at 36 to 37°C using an objective heater (TC-HLS-05, Bioscience Tools, San Diego, California, United States) and by applying direct current (DC) through insulated nichrome wires (Ace Glass, Vineland, New Jersey, United States) wrapped around the headplate holder (MAG-A, Narishige Group). The temperature was monitored using a microprobe thermometer (BAT-12, Physitemp Instruments, Clifton, New Jersey, United States).

To prepare for awake imaging, mice were habituated by being placed in the headplate holder and under the microscope objective for progressively longer durations (10, 20, 30, 45, 60, and 90 min) on consecutive days. They received sweetened milk as a reward every 30 min during both the training and imaging sessions.

Oxyphor 2P was initially prepared at a stock concentration of 80  μM in PBS and was diluted to 20  μM prior to infusion. We infused Oxyphor 2P at 0.05  μL/min, for a total volume of 1  μL, and then infused 0.5  μL of PBS. To generate an angiogram, 100  μL of 12.5  mg/mL fluorescein dextran, 70 kDa, was injected intravenously. Imaging was performed 2 h after the infusion. Both Oxyphor 2P and fluorescein dextran were excited at 950 nm. Phosphorescence intensity images (only one laser excitation cycle per pixel) were acquired at 250×250 resolution, and fluorescent angiograms were acquired at 512×512 resolution. Phosphorescence lifetime data were acquired using 2000  cycles/pixel and in a 20×20 grid. The lifetime of the phosphorescence of Oxyphor 2P was obtained by fitting the phosphorescence decay to a single-exponential function. The lifetime was converted to ${\mathrm{pO}}_{2}$ using a set of parameters determined in independent calibrations.^18^

### 2.7 Immunofluorescence

Mice were euthanized through ${\mathrm{CO}}_{2}$ inhalation and transcardially perfused with 25 mL of PBS followed by 25 mL of 4% paraformaldehyde (PFA) using a peristaltic pump (VWR International, Radnor, Pennsylvania, United States). The brains were extracted and kept in a fixing solution (4% PFA and 15% glycerol in PBS) overnight. Next, the brains were dehydrated in sucrose gradients of 10%, 20%, and 30%. After dehydration, the brains were sliced into 40-μm coronal sections using a sliding microtome (Leica SM2010R), and the sections were stored in 30% glycerol at −20°C until further processing for immunohistochemistry. The sections were subsequently permeabilized with Triton X-100, blocked with normal goat serum and incubated for 18 h with primary antibody for ionized calcium-binding adapter molecule 1 (IBA1) (rabbit polyclonal anti-IBA1, 1:1000, FUJIFILM Cat# 019-19741, RRID:AB_839504) at 4°C. The sections were washed with PBS and incubated with the secondary antibody (Alexa Fluor 647, 1:500, Molecular Probes Cat# A-21244, RRID:AB_2535812) for 1 h at room temperature. Next, the sections were incubated with glial fibrillary acidic protein (GFAP) antibody (mouse monoclonal anti-GFAP, 1:10000, Sigma-Aldrich Cat# C9205, RRID:AB_476889) for 1 h at room temperature. After washing, the sections were cover slipped with an antifade mounting medium with DAPI (H-1500, Vector Laboratories, Newark, California, United States) and imaged by confocal microscopy (Olympus FV3000).

## 3 Results

### 3.1 Development of Custom Cannula, Holder, and Procedure for Shallow-Angle Implantation

**Fig. 1:**
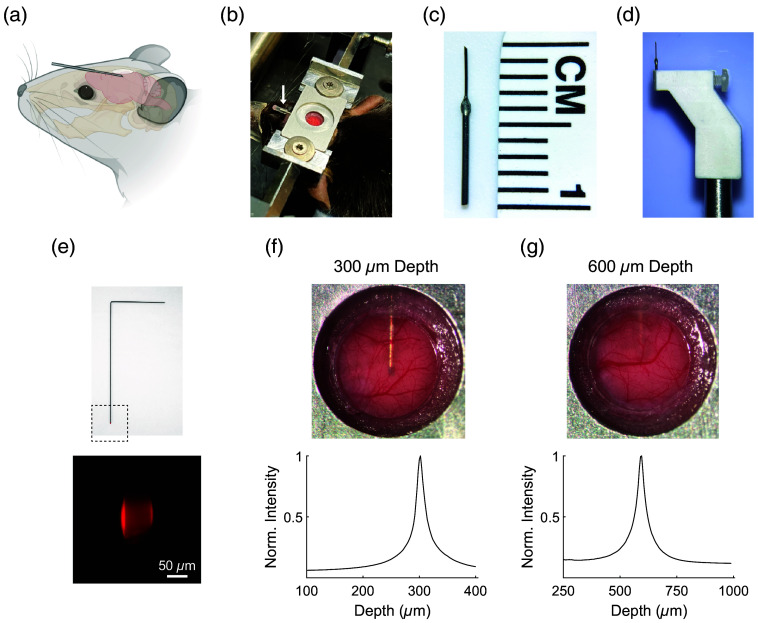
Design and characterization of shallow-angle cannula delivery system. (a) Schematic showing the position and the shallow approach angle of the implanted cannula in the mouse brain. (b) Image showing implanted cannula in a C57BL/6J mouse. White arrow indicates the location of the implanted cannula. The cannula tip is sealed by an attached segment of polyethylene tubing with a mixture of dental acrylic and cyanoacrylate glue at its end. (c) Cannula is created by combining segments of 26- and 33-G stainless steel tubing. (d) Custom cannula holder secures the cannula during implantation. (e) Fluorescent dummy wire is created to determine the depth of the implanted cannula. The length of the dummy wire is matched to the length of the cannula with the tip of the wire coated with SR101. Images of the cranial window after cannula implantation at 300  μm (f) and 600  μm (g) depths. The cannula is clearly visible at 300  μm depth, and a faint shadow of the cannula is visible at 600  μm depth. To confirm the location of the cannula tip, the fluorescent dummy wire was inserted into the cannula, and multiphoton microscopy was used to determine the depth-dependent fluorescence intensity profile.

Our objective was to develop a cannula-based delivery method for repeated infusion into the mouse cortex while maintaining the ability to perform *in vivo* multiphoton microscopy through a cranial window. This combined cannula and cranial window system needed to meet several criteria: (1) The cranial window needed to be of adequate size (4 mm) to image a relatively large portion of the underlying cortex. (2) The cannula’s tip had to be centered relative to the cranial window. (3) The cannula’s outlet had to be within the superficial to deep layers of the cortex, which are regions that are directly imageable by multiphoton microscopy. These requirements necessitated designing a low-profile cannula that could be implanted at shallow angles relative to the brain surface, ensuring the cannula would not obstruct the placement of the cranial window cover glass [Figs. 1(a) and 1(b)].

The cannula was created by combining segments of 26- and 33-G stainless steel tubing [Fig. 1(c)]. The portion of the cannula to be implanted inside the brain tissue was chosen to be 33-G to minimize impact on the brain tissue. A 45-deg bevel was added to the cannula tip to facilitate puncturing of the dura during implantation. Due to the fragility of the 33-G tubing, the portion of the cannula outside the brain was reinforced by the larger diameter 26-G tubing. A custom holder was created to securely hold the 26-G portion of the cannula [Fig. 1(d)]. The dimensions of the holder were chosen so that the holder would not make contact with the mouse head during the implantation process.

We tested two depths for cannula implantation: 300 and 600  μm. At 300  μm depth, corresponding to an implantation angle of 8 deg, the entire outline of the cannula up to its tip was visible in the cranial window image [Fig. 1(f)]. At 600  μm depth, corresponding to an implantation angle of 16 deg, only a shadow of the cannula was visible at the top of the cranial window [Fig. 1(g)]. We devised a method to verify the depth of the cannula after implantation by creating fluorescent dummy wires made of tungsten [Fig. 1(e)]. The dummy wires, matched in length to the cannula, had tips coated with the red fluorophore, sulforhodamine 101 (SR101). After inserting the dummy wire into the implanted cannula, we imaged the fluorescence in a volume surrounding the cannula tip. By plotting the fluorescence intensity as a function of depth, we could confirm the accurate localization of the cannula tip for both 300 and 600  μm depths [Figs. 1(f) and 1(g)].

We evaluated the neuroinflammatory response to the chronically implanted cannula in the brain by immunostaining for microglia and activated astrocytes in both the ipsilateral cortical region (right hemisphere, site of cannula implantation, and cranial window) and the contralateral cortical region (left hemisphere, control). To better understand the direct effect of cannula implantation, we also evaluated neuroinflammation in mice with cranial windows alone. At 30 days post-surgery, cannula-implanted mice showed a localized increase in microglia and astrocyte activation directly proximate to the location of the cannula and sparse astrocyte activation in other areas of the ipsilateral hemisphere, away from the cannula site [Fig. S1(b) in the Supplementary Material]. In cranial window mice, we also observed sparse activation of astrocytes in the ipsilateral hemisphere [Fig. S1(a) in the Supplementary Material]. We did not observe activated astrocytes in the contralateral hemisphere for either the cannula or cranial window mice. We performed quantitative analysis of microglia and activated astrocyte density in regions excluding the cannula site. For microglia density, we found that there were no significant differences between the ipsilateral and contralateral hemispheres in both the cranial window and cannula-implanted mice [Fig. S1(c) in the Supplementary Material]. Furthermore, a comparison of activated astrocyte density in the ipsilateral hemisphere between cranial window alone and cannula mice also showed no significant differences [Fig. S1(d) in the Supplementary Material].

### 3.2 Infusion and Imaging of Various Cell Type–Specific Markers, Structural Markers, and Markers for Alzheimer’s Disease Pathology in the Mouse Brain

We validated the ability of our approach to label and image various cellular targets *in vivo* by infusing a wide assortment of fluorescent cell markers and imaging with multiphoton microscopy. Our tests included both general cellular imaging markers and markers for imaging pathological processes in the brain. We specifically focused on fluorescent markers known to be challenging to introduce into the brain after systemic delivery due to their inability to cross the BBB. Many of the fluorophores we tested have been extensively used for *in vitro* live cell imaging experiments but previously have limited application for *in vivo* brain imaging.

**Fig. 2:**
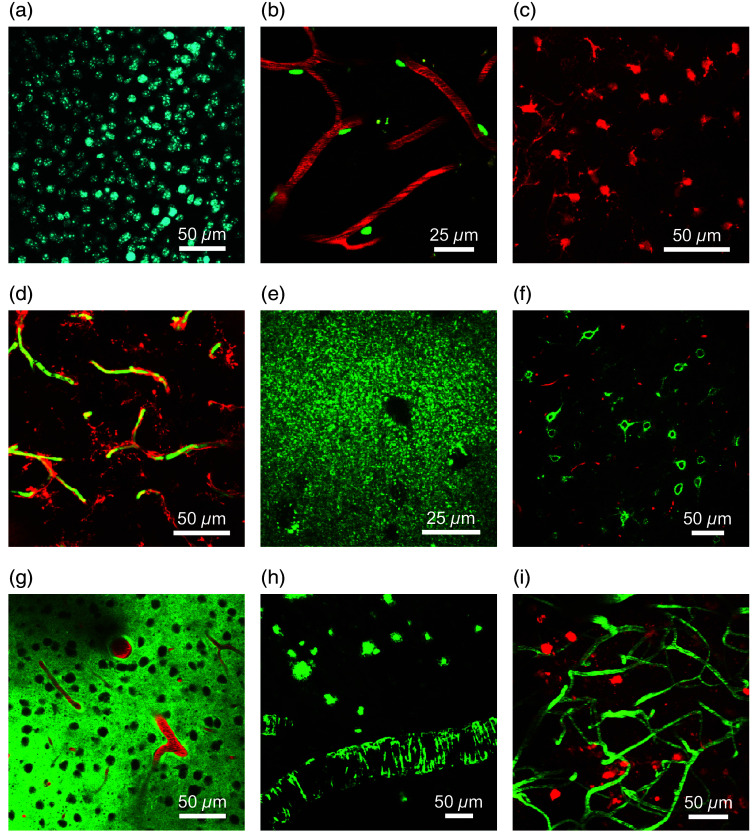
Validation of cannula delivery system using various fluorescent cell markers and probes for AD pathology. Fluorophores were infused through the cannula system and multiphoton microscopy was used for *in vivo* imaging. Images are shown as maximum intensity projections of 3D volumes. Each fluorescent cell marker was delivered to n=3 C57BL/6J mice. (a) Cell nuclei were labeled after infusion of Hoechst 33342. (b) Pericytes (green) were labeled after infusion of NeuroTrace 500/525 and rhodamine B dextran (red) was injected IV to act as an angiogram. (c) Astrocytes were labeled following the infusion of SR101. (d) Microglia (red) were labeled after infusion of Isolectin GS-IB4, Alexa Fluor 594 Conjugate. The vasculature was labeled using an IV injection of fluorescein dextran (green). (e) Lysosomes were labeled after infusion of LysoTracker Green DND-26. (f) Perineuronal nets (green) were labeled after infusion of fluorescein-labeled WFL and rhodamine B dextran (red) was injected IV. (g) Fluorescent Aβ42 (green) was infused to demonstrate perivascular clearance out of the brain (n=3 C57BL/6J mice). Rhodamine B dextran was injected to label the vasculature (red). (h) ThioS was infused and showed labeling of amyloid plaques and CAA in APP/PS1 mice (n=3). (i) Thiazine Red was infused in rTg4510 mice (n=3) and showed labeling of neurofibrillary tangles (red). Fluorescein dextran (green) was injected IV to serve as an angiogram.

We infused a nuclei acid binding fluorophore, Hoechst 33342, and observed bright and dense labeling of nuclei in the brain region surrounding the cannula outlet location [Fig. 2(a)]. Following infusion of NeuroTrace 500/525^24^ and intravenous (IV) injection of fluorescein dextran, we could detect labeled pericytes in close proximity to the blood vessels [Fig. 2(b)]. We could successfully image astrocytes [Fig. 2(c)] and microglia [Fig. 2(d)] after infusion of SR101^25^ and Isolectin GS-IB4, Alexa Fluor 594 Conjugate,^26^ respectively. We could visualize and track individual lysosomes after infusion of LysoTracker Green DND-26 into the brain [Fig. 2(e)]. By infusing fluorescein-labeled *Wisteria floribunda* lectin (WFL), we specifically targeted PNNs which are extracellular structures surrounding inhibitory neurons.^27^ Using this approach, we found dense labeling of PNNs throughout the cannula outlet area [Fig. 2(f)].

To demonstrate the utility of our approach for studying disease models in the brain, we also tested the infusion of fluorophores that could be applied for Alzheimer’s disease (AD) imaging. We explored the distribution of the amyloid-β (Aβ) peptide in the brain by direct infusion of fluorescently labeled Aβ42 peptide (HiLyte Fluor 488, Aβ42) into the mouse cortex. Rhodamine B dextran was systemically injected to create an angiogram. In regions above the infusion location, we observed an accumulation of fluorescent Aβ around the arterioles and arteries [Fig. 2(g)]. This perivascular fluorescence is consistent with previous reports after acute intracranial injection of fluorescent tracers.^28^ In addition, we tested our technique for imaging AD pathology in two mouse models of AD. As a model of cerebral amyloidosis, the APP/PS1 mouse model was used, which develops amyloid pathology starting at 5 to 6 months of age.^23^ For the labeling of pathology, we tested thioflavin S (ThioS), a fluorescent dye considered the gold standard for *ex vivo* labeling of amyloid aggregates.^29^ After infusing ThioS in APP/PS1 mice, we observed strong *in vivo* labeling of amyloid plaques and cerebral amyloid angiopathy (CAA) in the brain [Fig. 2(h)]. We used the rTg4510 mouse model as a model of tauopathy, which starts the development of NFTs at 2 months of age.^22^ Following infusion of Thiazine Red, a fluorescent dye known to bind to NFTs *ex vivo*,^30^ we found that we could successfully label and image NFTs in the brains of rTg4510 mice [Fig. 2(i)].

### 3.3 Longitudinal Tracking of Neurodegeneration in a Mouse Model of Alzheimer’s Disease

**Fig. 3:**
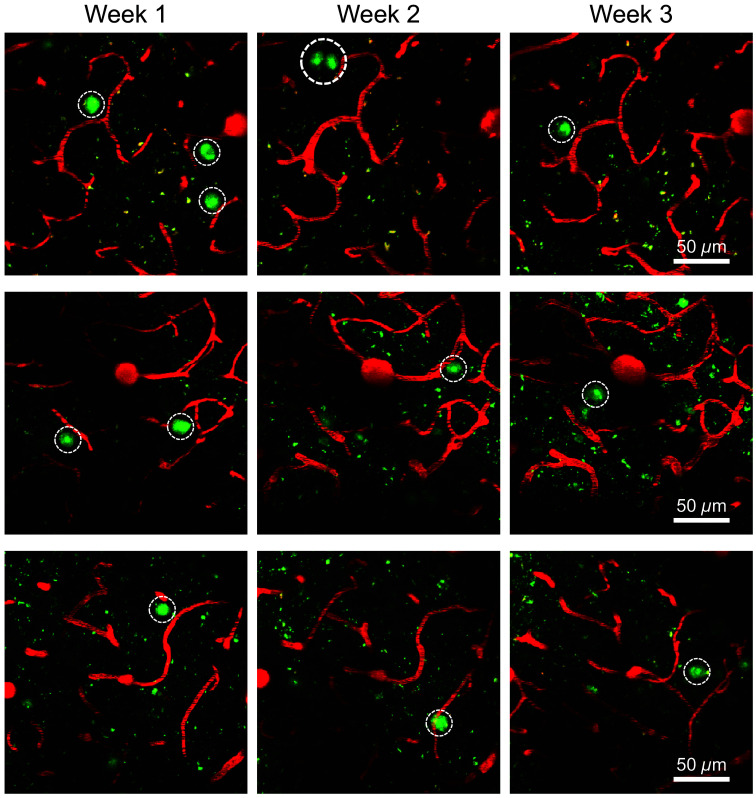
Longitudinal imaging of degenerating cells using Fluoro-Jade C in rTg4510 mice. Fluoro-Jade C (FJC, green) was infused through the cannula system on a weekly basis in rTg4510 mice (n=3). The cannula was implanted at 300  μm depth. Rhodamine B dextran (red) was systemically injected to create a fluorescent angiogram. Representative images show the appearance and disappearance of FJC-positive cells during weekly imaging (FJC-positive cells circled in white). Each row shows images from a unique mouse.

We next used our cannula-based delivery method to perform longitudinal imaging in the mouse brain. Our aim was to develop a technique to track neurodegeneration in real time in the mouse brain. To identify degenerating neurons, we used the fluorescent dye, Fluoro-Jade C (FJC) which has traditionally been applied for the staining of tissue sections. We used rTg4510 mice to model neurodegeneration, as neuron loss in the cortex of these mice has been well-documented.^22^ We infused FJC weekly for 3 weeks and conducted imaging with multiphoton microscopy. Prior to each imaging session, we injected IV rhodamine B dextran to form a fluorescent angiogram. We used the vascular structure from the angiogram as a reference to acquire the same fields of view on a weekly basis. After infusion of FJC into the transgenic mice, we found cells labeled by FJC scattered throughout the brain (Fig. 3). When comparing labeled cells in the same field of view, we found a dynamic process where cells labeled 1 week were mostly unlabeled the following week. In addition, regions without labeled cells in 1 week sometimes displayed new labeled cells in the subsequent week. When FJC was infused in age-matched control mice, we observed no FJC-labeled cells in the brain (Fig. S2 in the Supplementary Material). Our results indicate that our approach can successfully track neurodegeneration in a longitudinal manner in transgenic mice.

### 3.4 Longitudinal Imaging of Tissue ${\mathrm{pO}}_{2}$ in the Awake Mouse Cortex

We next applied our technique for longitudinal imaging to the measurement of tissue ${\mathrm{pO}}_{2}$ in the brain. For measuring ${\mathrm{pO}}_{2}$ *in vivo*, we used the two-photon excitable phosphorescent oxygen sensor Oxyphor 2P.^18^ The measured phosphorescence lifetime of the sensor can be directly mapped to the oxygen concentration in the brain. We infused Oxyphor 2P through our cannula delivery system on a weekly basis and collected two-photon phosphorescence emission data using a custom microscope. Fluorescein dextran was injected IV to act as an angiogram. Weekly imaging was performed in awake mice. The imaging field was focused around diving arteries in the brain, where we expected to observe the highest contrast in ${\mathrm{pO}}_{2}$ values. Phosphorescence lifetimes were measured within a ∼210-μm region of the ∼350-μm imaging field at points in a 20×20 grid.

**Fig. 4:**
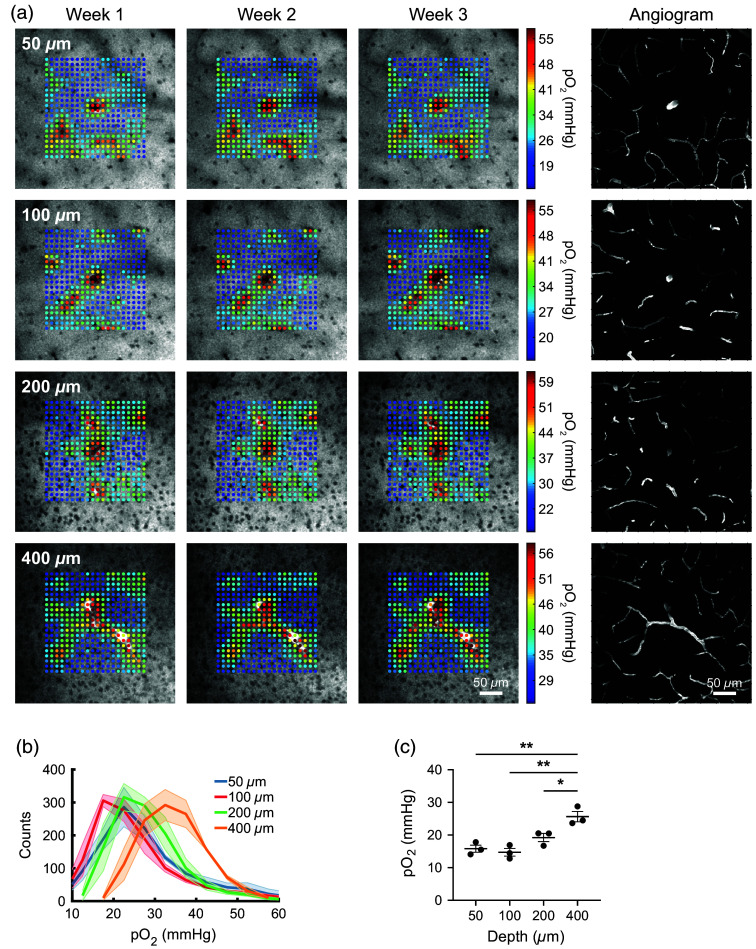
Longitudinal imaging of brain tissue ${\mathrm{pO}}_{2}$ in awake mice. Oxyphor 2P was infused through the cannula system on a weekly basis in C57BL/6J mice (n=3). The cannula was implanted at 600  μm depth. Imaging was performed using multiphoton phosphorescence lifetime microscopy. During the first weekly imaging session, the field of view was selected around diving arteries. (a) Representative images show color-coded ${\mathrm{pO}}_{2}$ values overlaid on top of phosphorescence intensity images. Images were acquired at 50, 100, 200, and 400  μm from the brain surface. Angiograms were acquired after IV injection of fluorescein dextran. The consistency and high contrast of the images from week to week demonstrate the viability of our approach for longitudinal ${\mathrm{pO}}_{2}$ imaging. (b) Histograms of ${\mathrm{pO}}_{2}$ values at each depth from n=3 C57BL/6J mice. The ${\mathrm{pO}}_{2}$ values are based on the average of measurements from all three weeks. (c) Quantification of the bottom 20% of the ${\mathrm{pO}}_{2}$ distribution (averaged over three weeks) at each depth from n=3 mice. Significance was calculated using one-way ANOVA with Tukey’s multiple comparison test: *P<0.05 and **P<0.01.

After infusion of Oxyphor 2P, we obtained high-contrast phosphorescence images of the probe down to 400  μm from the surface of the brain [Fig. 4(a)]. Color-coded ${\mathrm{pO}}_{2}$ levels (in mmHg) were overlaid onto the phosphorescence intensity image at each grid point. Cell bodies were clearly visible in the intensity images due to the negative contrast they provided in the images. The consistently sharp images of the cells over time indicated that the spatial resolution of the imaging was not compromised by repeated infusion. As expected, we found elevated ${\mathrm{pO}}_{2}$ in regions close to the diving arteries and its branches and more uniform reduced ${\mathrm{pO}}_{2}$ values in surrounding regions. We also found that the measured ${\mathrm{pO}}_{2}$ spatial maps were consistent on a week-to-week basis, indicating that our infusion technique is viable for longitudinal tracking of ${\mathrm{pO}}_{2}$. Furthermore, we demonstrated that our technique allows imaging of ${\mathrm{pO}}_{2}$ in anesthetized mice (Fig. S3 in the Supplementary Material). In anesthetized mice, we generally observed higher ${\mathrm{pO}}_{2}$ values both close to the diving arteries and surrounding regions compared with awake mice. We attribute this observed difference in ${\mathrm{pO}}_{2}$ levels to a reduction in local oxygen consumption in anesthetized mice. To determine the dependence of ${\mathrm{pO}}_{2}$ on depth in awake mice, we obtained the histogram for each depth and found that there is a shift of the ${\mathrm{pO}}_{2}$ distribution toward higher ${\mathrm{pO}}_{2}$ values at larger depths [Fig. 4(b)]. In addition, we measured the average of the bottom 20% of the ${\mathrm{pO}}_{2}$ distribution, corresponding to regions of the field of view away from the diving artery [Fig. 4(c)]. We found an increase in the bottom 20% of the ${\mathrm{pO}}_{2}$ distribution for larger depths, which is consistent with a recent study measuring the depth dependence of ${\mathrm{pO}}_{2}$ in awake mice at a single time point.^31^

## 4 Discussion and Conclusion

We have developed a cannula-based system that enables intracranial delivery into the mouse brain and is fully compatible with *in vivo* multiphoton microscopy. The basis of our technique is that to achieve targeting of the superficial mouse cortex (∼300  μm) while keeping the cannula outlet centered relative to the cranial window necessitates a very shallow angle of approach. This led to the development of a low-profile cannula and an implantation procedure that is compatible with the shape of the mouse head. Importantly, as the angle of the cannula relative to the brain surface is nearly parallel, the cannula does not obstruct the imaging objective for multiphoton microscopy. This is in contrast to other cannula-based approaches for brain delivery which use bulkier commercial cannulas that occupy more space above the brain and are limited in how shallow the implantation angle can be.

As the cannula in our approach is permanently implanted, it can bypass some of the issues associated with using soft, penetrable materials either as ports for the injection or for the entire cranial window. In these approaches, an injection needle or micropipette is inserted each time for brain delivery. This has the potential to puncture the blood vessels and create new tracts of tissue damage during each injection. This issue is especially significant when targeting deeper regions of the brain. In addition, the location for each injection may differ, depending on the injection accuracy. In our cannula-based technique, the damage to the brain occurs once during the initial implantation. Also, infusion using the cannula is highly reproducible, as the outlet location of the cannula is stable. This ensures that the infused substance exposes the same brain regions at the same dosage, which makes comparisons after each infusion more straightforward.

Our technique may also offer certain advantages over traditional acute intracranial injections. In our approach, the cannula outlet is below the area of imaging, whereas for acute injections, the damage occurs from the brain surface down to the depth of the injection with the damaged region located centrally relative to the imaging area. Another key difference is that with our approach the infusion is performed when the brain has already recovered from the cannula implantation. In contrast for acute injections, the injection is performed immediately after the needle is inserted into the brain. This difference may influence the brain’s inflammatory state and its reaction to the delivered substance, particularly in the context of delivering viral vectors.

When we conducted pilot tests on our cannula-based system by infusing various imaging agents, we found that the infusion would sometimes cause neuroinflammation and infiltration of immune cells into the brain, especially near the top of the brain. This would lead to the cranial window becoming more opaque and would ultimately limit the achievable depth of imaging. We found that we could frequently mitigate this issue by reducing the concentration of the imaging agent. Future studies need to be conducted to understand why certain chemical compounds trigger a response whereas others do not and how this response is influenced by infusion parameters including volume, concentration, and rate.

We believe our developed technique will enable many new types of longitudinal imaging studies in the brain. So far, we have successfully demonstrated the tracking of neurodegeneration and the measurement of brain tissue partial pressure of oxygen over time. Given the wide assortment of imaging agents that are commonly used *in vitro* for live cell imaging, which have previously never been applied for *in vivo* imaging with multiphoton microscopy, we believe our approach should allow the testing of many of these agents *in vivo* and enable their application for longitudinal imaging. In addition, our technique should have utility in preclinical imaging studies for the repeated delivery and imaging of therapeutic agents in the brain.

## Supplementary Material

10.1117/1.NPh.12.2.025001.s01

## Data Availability

The data that support the findings of this study are available from the authors upon reasonable request. See author contributions for specific datasets.
